# Cognitive and Motor Learning in Internally-Guided Motor Skills

**DOI:** 10.3389/fpsyg.2021.604323

**Published:** 2021-04-09

**Authors:** Krishn Bera, Anuj Shukla, Raju S. Bapi

**Affiliations:** Cognitive Science Lab, Kohli Center on Intelligent Systems, International Institute of Information Technology, Hyderabad, India

**Keywords:** motor sequence learning, skill learning, internally-guided sequencing, grid-navigation tasks, cognitive learning

## Abstract

Several canonical experimental paradigms (e.g., serial reaction time task, discrete sequence production task, *m* × *n* task) have been proposed to study the typical behavioral phenomenon and the nature of learning in sequential keypress tasks. A characteristic feature of most paradigms is that they are representative of *externally-specified* sequencing—motor tasks where the environment or task paradigm extrinsically provides the sequence of stimuli, i.e., the responses are stimulus-driven. Previous studies utilizing such canonical paradigms have largely overlooked the learning behaviors in a more realistic class of motor tasks that involve *internally-guided* sequencing—where the sequence of motor actions is self-generated or internally-specified. In this work, we use the grid-navigation task as an instance of internally-guided sequencing to investigate the nature of learning in such paradigms. The participants performed Grid-Sailing Task (GST), which required navigating (by executing sequential keypresses) a 5 × 5 grid from start to goal (SG) position while using a particular key-mapping (KM) among the three cursor-movement directions and the three keyboard buttons. The participants performed two behavioral experiments—Single-SG and Mixed-SG condition. The Single-SG condition required performing GST on a single SG position repeatedly, whereas the Mixed-SG condition involved performing GST using the same KM on two novel SG positions presented in a random, inter-mixed manner. In the Single-SG condition, we show that motor learning contributes to the sequence-specific learning in GST with the repeated execution of the same trajectories. In the Mixed-SG condition, since the participants utilize the previously learned KM, we anticipate a transfer of learning from the Single-SG condition. The acquisition and transfer of a KM-specific internal model facilitates efficient trajectory planning on novel SG conditions. The acquisition of such a KM-specific internal model amounts to trajectory-independent cognitive learning in GST. We show that cognitive learning contributes to the learning in GST by showing transfer-related performance improvements in the Mixed-SG condition. In sum, we show the role of cognitive and motor learning processes in internally-guided sequencing and further make a case for using GST-like grid-navigation paradigms in investigating internally guided skill learning.

## Introduction

Our everyday experiences are an excellent demonstration of the surprisingly adaptive and fluid learning behavior that is orchestrated by the human brain. Such a learning behavior is a hallmark of human cognitive ability and spans a broad spectrum of tasks. Ranging from complex tasks such as cycling and driving to seemingly simpler ones such as typing and grasping movements, all tasks involve the acquisition of skillful behavior. Skill learning is a natural behavioral phenomenon concerned with the acquisition of the ability to perform tasks proficiently. *Motor skill learning* refers to learning a specific subclass of skills that involve sequential motor movements such that they are executed accurately and quickly with practice (Newell, 1991; Clegg et al., 1998; Haibach et al., 2017; Schmidt et al., 2019). Much of the early interest in motor sequencing focused on investigating the typical behavioral phenomenon in sequence learning tasks (Lashley, 1951; Hebb, 1961; Fitts and Posner, 1967). This has led to the formulation of many serial order canonical experimental tasks such as the *m* × *n* task (Hikosaka et al., 1995; Bapi et al., 2000, 2006) and discrete sequence production (DSP) task (Verwey, 2001; Abrahamse et al., 2013; Verwey et al., 2015) in the explicit domain and serial reaction time (SRT) task (Nissen and Bullemer, 1987; Willingham, 1999; Robertson, 2007) in the implicit domain. While explicit learning involves conscious awareness of what is being learned, implicit learning occurs without conscious awareness of learning. Subsequent research has extensively used these paradigms to understand the brain processes involved in sequence learning, memory, attention, etc.

In SRT and DSP tasks, the participants repeatedly respond to a fixed set of visual stimuli organized in successive trials. Each trial involves presenting a sequence of visual cues that prompt corresponding keypress responses on a visuospatially-compatible button-box. In the *m* × *n* task, each trial consists of *n* consecutive visual stimuli (called a hyperset). Each visual stimulus consists of *m* illuminated squares on a 3 × 3 grid presented on a screen. The participants learn to press *m* corresponding keys (called a set) successively in the correct order on a keypad in response to the visual stimulus. The visual stimuli that guide the sequencing behavior in such paradigms are predetermined and fixed by experimental design. The sequence of motor actions to be performed is not contingent on the participant’s choice or plan. Therefore, these canonical tasks belong to a class of discrete sequence learning tasks that involve *externally-specified* (visual) sequences. The sequence of motor actions in such tasks is conditioned on fixed, externally-specified visual cues/stimuli.

While such simple canonical paradigms are useful for investigating skill learning in controlled experimental settings, they fail to account for a larger class of real-life motor tasks. Unlike SRT, DSP, or *m* × *n* task, many real-life motor skills are internally-guided, i.e., the sequence of the motor actions is triggered by self-choice or some internal model of the environment. Such tasks constitute a class of *internally-guided* motor tasks. The sequence of actions is self-initiated or generated internally by the participant and is not extrinsically prescribed or predetermined by the environment. Unlike externally-specified sequencing, the sequential action in such tasks is not elicited as a chain of stimulus-response pairs. While the visual cues might help the agent make sense of the environment in such tasks, it does not specify the sequence of motor movements to be executed. The central point of difference between externally-specified and internally-guided sequencing is that the latter involves volitional planning of motor action sequences. A template tracing task on paper is an example of an externally-specified task. It employs external cues and visual feedback with a greater role of visuomotor associations for imitating the given template. On the other hand, drawing is an internally-guided task that relies on internal cues for guiding the pencil strokes to self-determined positions on paper. Such behavior is characterized by greater demands on brain processes related to memory and planning as compared to the tracing task. Other examples of such motor skills are composing music on a keyboard, creating a dance choreography, or solving a Rubik’s cube. Such tasks involve planning as well as execution of a self-generated sequence of motor actions. The performance in internally-guided sequencing tasks depends on the dexterity of executing the motor actions and the ability to program the sequence of future actions.

Previous studies have investigated the motor behavior in externally-guided and internally-guided tasks and determined the neural underpinnings of the underlying processes. The externally-guided movements predominantly involve brain areas related to sensory guidance and optimization of movements, perception, and salience, whereas internally-guided movements involve brain areas related to muscle/movement selection, mental imagery, and planning complex behaviors (Gowen and Miall, 2007; Drucker et al., 2019). Other investigations have confirmed the role of cerebellar and premotor circuits in externally-guided tasks and basal ganglia, pre-supplementary motor cortex and dorsolateral prefrontal cortex in internally-guided tasks (Jueptner et al., 1996; Jueptner, 1998; van Donkelaar et al., 1999).

In externally-specified sequencing, bindings between the presented stimuli and the corresponding responses emerge with simple association rules between stimuli and response (S-R): selecting an action in response to a given stimulus binds the codes of the action-relevant stimulus attributes and the corresponding action codes (Logan, 1988). Due to repeated execution of sequences, the activity of the system controlling stimulus-based actions results in stimulus-response or sensorimotor learning (Herwig and Waszak, 2009). Therefore, the sequencing in the externally-specified domain is exhibited as a chain of stimulus-response-effect (S-R-E). On the other hand, the internally-guided or voluntary actions typically involve a goal-directed motivation to achieve an internally pre-specified outcome. The studies have shown that such self-determined action goals play a role in the acquisition and planning of internally-guided actions (Hommel et al., 2001; Hommel, 2003). The activity of the system guiding intention-based actions results in action-effect or ideomotor learning due to the formation of associations between movements and their ensuing sensory effects (Herwig and Waszak, 2009). According to the ideomotor framework of action control (Greenwald, 1970; Prinz, 1997), internally-guided actions primarily refer to anticipated action effects or, in other words, response-stimulus (R-S) bindings. In internally-guided actions, the participants might only attend to response-effect (R-E) contingencies (Herwig and Waszak, 2009). None of the previous studies have explored the nature of learning processes in such a class of discrete, self-guided sequential movement tasks. Motivated by this apparent gap, our present study investigates the role of different learning components in internally-guided sequencing.

Sequence learning in simple grid-navigation tasks is an example of an internally-guided sequencing task. The tasks involve navigating (typically, using a cursor) on the grid from the *start* position to the *goal* position. Each unique trajectory from the start to the goal position constitutes a novel sequence of keypresses. The optimality of trajectory is conditioned on the task specifications such as the reward scheme, possible agent movements, and time constraints. Participants are free to choose among many possible optimal trajectories for a trial to be successful. The repeated execution of these trajectories results in learning a self-generated, voluntary sequence of keypresses. The behaviors in grid-navigation tasks give us rich insights into the learning processes involved in internally-guided sequencing. We propose a novel usage of the simple grid-navigation task—*Grid-sailing task* (GST; Fermin et al., 2010, 2016) as a canonical paradigm to investigate the learning processes involved in internally-guided sequencing. The GST requires navigating a 5 × 5 grid from start to goal (SG) position using a given key-mapping (KM). The KM associates possible movement directions of the cursor with the corresponding keyboard buttons. The participants are instructed to reach the goal in an optimal number of steps as quickly as possible. Table 1 provides a concise summary of different sequencing tasks—SRT, DSP, *m* × *n*, and GST—based on the experimental paradigm and the nature of learning involved.

**Table 1:** Task comparison between externally-specified (SRT, DSP, *m* × *n*) and internally-guided sequencing tasks.

	Serial reaction time task (SRT)	Discrete sequence production task (DSP)	*m* × *n* task	Grid-sailing task (GST)
**Features of experimental paradigm**				
**Number of effectors**	1/2	2	1	1
**Number of choices/fingers used**	4	4/6/8	3	3
**Stimuli**	Visual: spatially-compatible and key-specific	Visual: spatially-compatible and key-specific	Visual: consists of *m* illuminated squares on a grid	Visual cues for start, goal and agent positions
**Sequence length**	10	3–8	10–12	5–7
**Number of trials**	800	500–1,000	10–20 successful trials	20
**Behavioral measures**	Response time	Response time	Choice time, movement time	Reward, number of moves, execution time, reaction time
**Nature of sequences and learning**				
**Sequence specification**	Explicitly specified	Explicitly specified	Explicitly specified—discovery by trial and error	Internally planned
**Kind of sequences learnt**	Typically first-order and second-order	Typically first-order and second-order	Hierarchical sequence	Higher-order trajectory of grid-cell states
**Nature of learning**	Implicit	Explicit	Explicit	Explicit

Specifically, we considered the involvement of two learning components—motor and cognitive. The cognitive component involves learning the sequential order of movements, whereas the motor component concerns the acquisition of fine-tuned movement dynamics and sensorimotor integration (Doya, 2000; Ghilardi et al., 2009; Penhune and Steele, 2012). Using GST as our canonical paradigm, we employ two behavioral experiments to identify the underlying learning processes in the internally-guided sequencing. In Experiment-1 (Single-SG condition), participants perform GST on a single SG-condition. We show evidence for motor learning due to the repeated execution of sequences. In Experiment-2, the participants use the learned KM from Experiment-1 to perform grid-navigation on the Mixed-SG condition, which consists of randomized trial order of two previously unseen SG conditions. A successful transfer of a KM-specific internal model would enable efficient trajectory planning on the novel SG conditions and, thus, would point out the role of the cognitive learning in Experiment-2. We further make a case for using GST-like grid-navigation tasks for investigating the typical behavioral phenomena in internally guided sequencing.

## Experiment-1: Single-SG Condition

We hypothesize that sequence-specific motor learning contributes to the learning in GST. As the participants repeatedly execute the same trajectory, the motor movements are optimized to facilitate accurate and fast sequential keypresses. This can be empirically tested by examining the effect of trials on the mean execution in Experiment-1 (also referred to as the Single-SG condition). The Single-SG condition also involved a rotation trial to test whether the learning in GST occurs due to the acquisition of a motor program or general motor improvements. The general motor improvements can result from factors such as task familiarity or adaptation. The rotation was introduced such that the sequence of keypresses required to navigate the cursor from the start position to the goal position remained the same as in the normal trials. Consequently, the execution time on the rotation trials is expected to remain unaffected if the performance improvements in GST occur only due to general motor improvements.

### Materials and Methods

#### Participants

Forty-two healthy participants volunteered for the study. The participant pool consisted of 29 women and 13 men between ages 17 and 27 (mean age: 21) years. All participants were non-musicians with normal or corrected-to-normal vision. The study was approved by the Institute Review Board, IIIT-Hyderabad, India. The participants gave informed written consent before the study. Additionally, permission for participation was obtained from the College Principal for participants below 18 years of age. The participants initially performed Experiment-1 (Single-SG condition with visuomotor rotation trial) followed by Experiment-2 (Mixed-SG Condition).

#### Apparatus

The participants were seated on a chair facing a high-resolution 24-in computer screen placed ~2 ft away. A conventional desk keyboard was used to record responses. The participants used the right index, middle, and ring fingers to press the number-pad buttons “4,” “5,” and “6,” respectively. All the other keys on the number-pad were removed to prevent meddling in response selection. The experiment program for stimulus presentation and data recording was written using Python3 and PyGame (Python Game Development^1^).

#### Procedure

**Figure 1:**
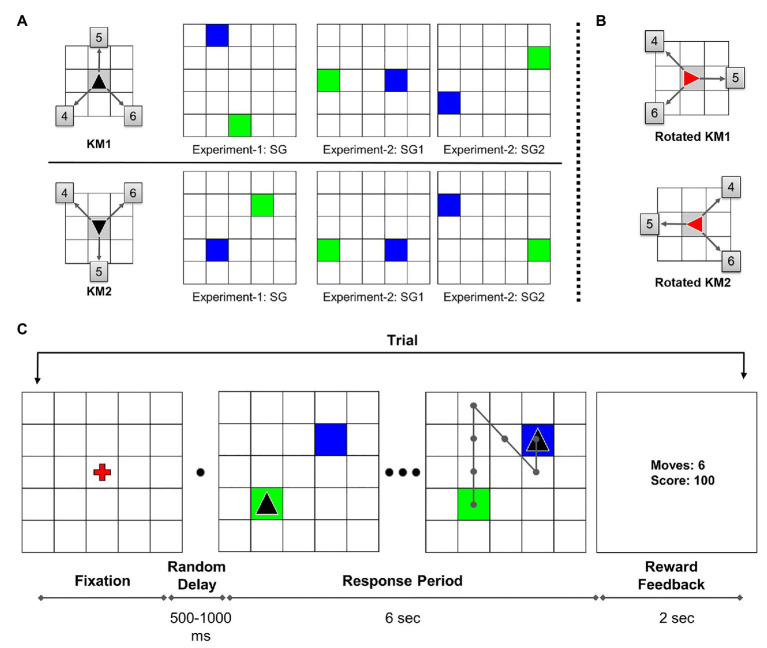
**(A)** Key-mapping (KM) and start-goal (SG) position sets used in the experiment. Each participant was randomly assigned either KM1 or KM2. The boxed numbers on KM figure show corresponding numeric keys associated with the movements. In SG figures, green and blue tiles represent start and goal positions, respectively. **(B)** The 90° clockwise rotated KMs used in the rotation trials in Experiment-1. **(C)** Task diagram: sequence of trial events (adapted from Fermin et al., 2010). In this illustration, the participant is assigned key-map KM1. An example optimal trajectory is shown on the grid.

The participants were verbally instructed about the task procedure before the session started. A 5 × 5 grid with a red fixation cross at the center was displayed at the beginning of each trial. On pressing the “space” button, after a random delay of 500–1,000 ms, the trial started with the start position marked as a green tile and the goal position marked as a blue tile. The cursor, shown as a black triangle, was initially placed in the starting position. The participants were given 6 s to solve each trial, and this duration was not explicitly conveyed to them. During the trial response period, participants executed sequential keypresses to navigate the cursor from the starting position to the goal position. The possible cursor-movement directions were defined by the KM (see Figure 1A). In the beginning, the task required participants to explore the KM directions and its association with the corresponding keys by trial and error.

The participants were explicitly instructed to achieve a maximum score (of 100 points) while executing each trial as quickly as possible. If an optimal path is traversed, a maximum of 100 points is awarded for that trial. A minimum steps trajectory from start to the goal is considered an optimal trajectory. If the participant took a non-optimal path, a penalty of −5 points incurred for every excess move. In case the participant tried to perform an invalid move, such as moving out of the grid, the cursor position remained the same with an incremented move count. If the participant failed to reach the goal in the given time duration, 0 points were awarded for that trial. At the end of each trial, the performance feedback was displayed for 2 s, following which the fixation screen signaled the beginning of the next trial. On the center of the feedback screen, the performance feedback was presented as two numbers. The display showed the number of moves in the traversed trajectory and the reward score for that trial. A trial outline is shown in Figure 1C. The participants were given a rest block after every 20 trials to minimize the effects of muscle fatigue on the performance. The participants were also advised to maximally re-use the explored trajectories in order to execute the task quickly and accurately.

Two different KMs were used in the experiment to avoid any unwanted performance effects or bias due to a particular KM. Moreover, each KM was associated with a unique set of SG pairs (see Figure 1A). The participants were randomly assigned one of the two possible KMs. The participants used the same assigned KM throughout the experiment for both, Single-SG and Mixed-SG conditions. Twenty-four participants used KM1, whereas 18 participants used KM2 for the experiment.

In Experiment-1, the participants repeatedly performed GST on a single SG condition. The participants were presented with the same SG condition for trials 1–41. The rotation trial (trial 42) was introduced after the completion of 41 successful trials. The rotation trial was followed by the re-introduction of the learned single SG condition for the next five trials (trials 43–47). The post-rotation trials (trials 43–47) were used only for a comparative analysis between the rotation and normal condition. The rotation trial involved a 90° clockwise rotation of the grid. The start and goal positions also changed accordingly with the grid rotation. The rotated cursor changed its color from black to red to indicate the transformed KM associations (see Figure 1B). Therefore, the sequence of keypresses required to reach the goal position effectively remained the same. In the case of error trials in the rotation condition, the participants were repeatedly presented with the rotation trial. The participants were already instructed about the rotation trial beforehand. The participants took about 15 min to complete Experiment-1.

#### Behavioral Measures

The number of moves in the traversed trajectory, reward obtained, reaction time and execution time were the performance measures recorded for each trial of the experiment. Reaction time is defined as the time interval between the onset of stimuli and the first keypress. Execution time is the total time taken for sequential keypresses in a particular trial. Execution time is computed as the difference between the keypress time of the last and the first response. For analysis purposes, the trials were classified into three categories (1) Successful trials—if the goal position is reached with a non-zero reward, (2) Optimally successful trials—if the goal position is reached in an optimal number of moves and thereby scoring a maximum reward, and (3) Error trials—if the goal position is not reached in the given time duration.

### Results

The following behavioral measures were included for the analysis: reward score, reaction time, execution time, number of moves, and error rate. The error rate is a computed measure that denotes the average number of error trials attempted to complete one successful trial. The successful and error trials were both included in the analysis to show the emergence of learning and skillful behavior in the task. However, only successful trials were considered for other analysis purposes. A within-subjects repeated-measures ANOVA was used to test for the effect of practice (trials) on different behavioral measures. A series of Wilcoxon signed-rank tests was performed on various measures to compare the performance on rotation and normal trials. Repeated-measures ANOVA was used to probe any KM-specific effects on the performance. The statistical analysis was performed using Python (scipy and statsmodels packages) and JASP software (JASP Team, 2020).

**Figure 2:**
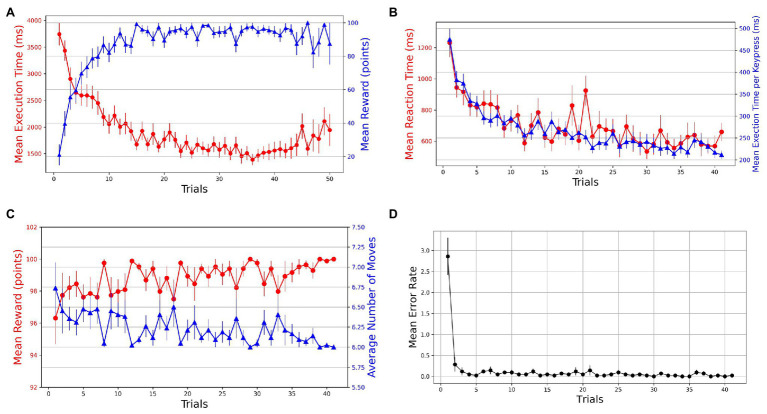
Trial-by-trial course of performance improvement in Single-SG condition (rotation trial excluded). The bars on the plot data-points denote standard error in measurement. **(A)** Evolution of learning behavior in the task. Mean execution time and mean reward across trials—averaged over both successful and error trials. **(B)** Mean reaction time and normalized execution time in successful trials. **(C)** Mean reward and average number of moves in successful trials. **(D)** Mean error rates in successful trials.

The learning in the task is evident from the performance improvements in various behavioral measures. With practice, we see an increasing and decreasing trend in reward and execution time, respectively, which suggests that within a few (10–15) trials, the participants progressively learned to perform the task while optimizing for speed (execution time) and accuracy of navigation (reward; see Figure 2A). We took reward, moves, execution time, and reaction time as dependent measures of learning for successful trials. To evaluate the learning behavior, we plotted the mean values of behavioral measures in successful trials (see Figures 2B,C). The mean reward increases to a maximum of 100 points as the number of moves reduces over the practice to reach the optimal/minimum number of steps. A non-parametric Friedman test of differences among repeated measures (within-subjects) rendered a significant effect of trials on average reward obtained [$\chi^{2}\left(40\right)=73.97,p<0.001$] and the average number of moves required to reach the goal [$\chi^{2}\left(40\right)=73.97,p<0.001$].

The learning is also evident by comparing the mean execution time of the first successful trial (*M* = 3,110 ms, SD = 1,062) with the last successful trial (*M* = 1,271 ms, SD = 380 ms). The mean reaction time decreased from 1,232 ms (SD = 592) to 659 ms (SD = 380). The Friedman test indicated significant improvements in execution time [$\chi^{2}\left(40\right)=485.90,p<0.001$] as well as reaction time [$\chi^{2}\left(40\right)=300.55,p<0.001$]. However, this decrease in execution time could have been a function of the number of moves in the trajectory. Therefore, we computed normalized execution times or execution time per keypress to account for the unequal lengths of trajectories in successful trials. The Friedman test indicated significant improvements in normalized execution time [$\chi^{2}\left(40\right)=499.93,p<0.001$]. The acquisition of learned sequences was examined by computing the error rates and plotting them against trials. A steep decrease in error rates is observed over the first few trials (see Figure 2D). Additionally, sequence-specific motor learning was examined by controlling the number of keypresses and the trajectories followed. For each participant, the most frequently used optimal trajectory was determined. The trials that employed the most-frequented optimal trajectory were extracted. A decrease in mean execution time from 2,473 ms (SD = 1,051) to 857 ms (SD = 157) in extracted trials suggests sequence-specific learning. To evaluate the performance improvements across these trials, we performed a Friedman test, which indicated a significant effect of trials [$\chi^{2}\left(38\right)=78.72,p<0.001$] on execution time.

In order to examine whether the learning observed in the GST was particular to KM, we performed 2 (KM: 1 and 2) × 41 (Trials: 1–41) mixed repeated-measures analysis of variance (ANOVA) on normalized execution time. The KM was used as a between-subject factor, and trials were a within-subject factor. A Greenhouse-Geisser correction was applied when the ANOVA assumptions were violated. The ANOVA results suggested a significant main effect of trials [$F\left(11.16,446.25\right)=19.148,p<0.001,\eta_{p}^{2}=0.324$] on the normalized execution times. Similarly, a significant main effect of KM [$F\left(1,40\right)=7.517,p=0.009,\eta_{p}^{2}=0.158$] indicated that the normalized execution times are different for the two KM. However, the Trial × KM interaction was not found to be significant [$F\left(11.16,446.25\right)=1.347,p=0.194,\eta_{p}^{2}=0.033$], suggesting that the variation in normalized execution time across the trials is not dependent on KM.

**Figure 3:**
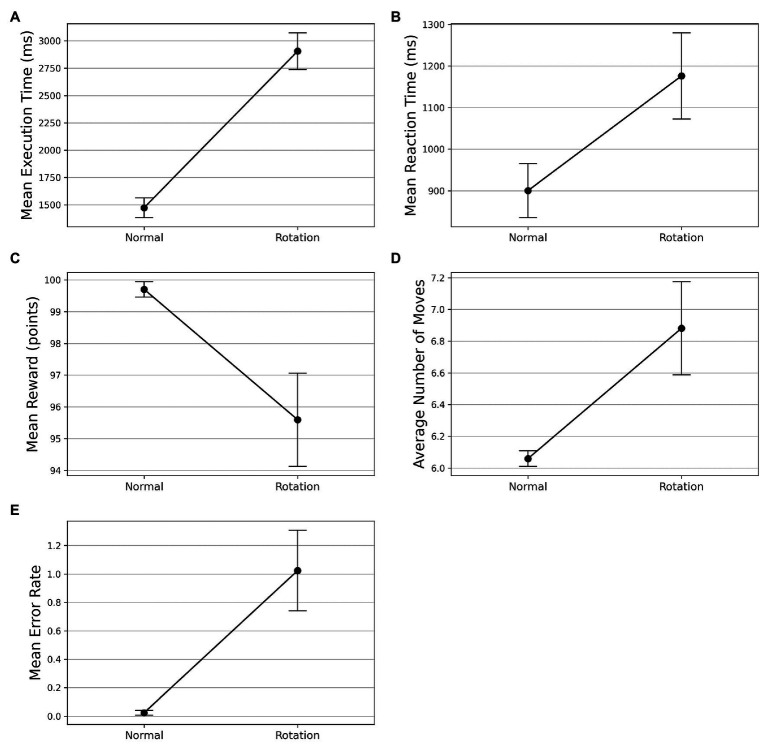
Comparison of performance on normal and visuomotor rotation trials in Experiment-1. The bars on the plot data-points denote standard error in measurement. **(A)** Mean execution time on optimally-successful rotation trials is significantly higher than the average of preceding and succeeding optimally-successful trials. **(B)** Mean reaction time on successful rotation trials is significantly higher than the average of preceding and succeeding successful trials. On the rotation trial, the average reward obtained **(C)** is significantly lesser while the average number of moves **(D)** is significantly higher. **(E)** The mean error rate also increases in the rotation trials.

On the visuomotor rotation trial (trial 42), we observed a spike in the execution time (see Figure 3A). The execution time comes down with the re-introduction of the learned SG condition after the rotation trial. To assess whether the mean execution time for the visuomotor rotation trial is significantly higher than the normal condition, we took the average execution time of the preceding and the succeeding optimally successful trials and compared it with the rotation trial (trial 42). The mean execution time increased from 1,473 ms (SD = 496) in the normal trials to 2,906 ms (SD = 919) in the rotation trial. A Wilcoxon signed-rank test was used as the normality assumptions were violated. It suggested that the mean execution time for the rotation trials is significantly higher than the normal trials (df=29,Z=0,p<0.001). Similarly, the mean reaction time increased from 900 ms (SD = 420) in the normal trials to 1,176 ms (SD = 672) in the rotation trial (see Figure 3B). The Wilcoxon signed-rank test indicated that the difference in mean reaction time on the rotation trial and normal trials was significant (df=41,Z=245.50,p=0.010). On following a similar procedure, we found that the mean reward obtained decreased from 99.702 (SD = 1.581) to 95.595 (SD = 9.513) on the rotation trials (see Figure 3C). The Wilcoxon signed-rank test suggested that the difference in mean reward score obtained on normal and rotation trials is significant (df=41,Z=96.50,p=0.006). Similarly, the number of moves executed increased from 6.060 (SD = 0.316) in the normal trials to 6.881 (SD = 1.903) in the rotation trials (see Figure 3D). The Wilcoxon signed-rank test suggested that the increase in the number of moves is significant (df=41,Z=8.50,p=0.006). The error rates also increased from 0.024 (SD = 0.108) to 1.024 (SD = 1.828) in the rotation trials (see Figure 3E). The Wilcoxon signed-rank test also suggested a significant difference in error rates (df=41,Z=0,p<0.001) in both conditions.

### Discussion

In line with previous GST studies and other skill learning tasks, the performance improvements in terms of speed (execution time, reaction time) and accuracy (reward) suggest the acquisition of skillful behavior (Nissen and Bullemer, 1987; Hikosaka et al., 1995; Willingham, 1999; Sakai et al., 2003; Fermin et al., 2010; Abrahamse et al., 2013). The practice-driven performance improvements in various behavioral measures in Single-SG trials suggest overall learning in GST. The practice-driven performance improvements in execution time provide evidence for motor learning that occurs due to the fine-tuning of motor movements. The Single-SG condition also included a visuomotor rotation trial to probe if the performance improvements in GST can be solely attributed to general motor improvements. The performance degradation on execution time and other measures such as reward, reaction time, and error rate suggests that the performance improvements in GST can be attributed to the sequence-specific learning processes (specifically, the acquisition of the motor program).

## Experiment-2: Mixed-SG Condition

In Experiment-1, the participants repeatedly performed GST on a single SG condition using the same KM. The participants not only learned the motor program associated with the sequence of movements to reach the goal position but also internalized the navigation strategies related to the specific KM. The results of Experiment-1 established trajectory-specific motor learning. Further, to investigate KM-specific cognitive learning, we designed Experiment-2 (also referred to as the Mixed-SG condition) to test the transfer of KM-specific learning to novel SG conditions. We anticipate that the transfer of KM-specific learning will lead to efficient trajectory planning on novel SG conditions. The account can be empirically tested by comparing various performance measures during the initial trials of Experiment-1 and Experiment-2.

### Materials and Methods

#### Participants

All the participants performed Experiment-2 after completing Experiment-1.

#### Apparatus

The experimental setup and apparatus were the same as in Experiment-1.

#### Procedure

In the Mixed-SG condition, the general task paradigm was the same as in Experiment-1 except for the SG-conditions. In the Mixed-SG condition, participants employed the previously learned KM (from Experiment-1) to perform grid-navigation on two novel SG conditions. The optimal number of steps in both the SG conditions were the same. During the experiment, each trial was randomly assigned to one of the two possible SG conditions. The participants performed GST on the randomized and mixed order of SG conditions. The experiment terminated when the participant performed 20 successful trials of each SG condition. The participants took about 15 min to complete the Mixed-SG condition task.

#### Behavioral Measures

The behavioral measures logged and analyzed were the same as in Experiment-1.

### Results

**Figure 4:**
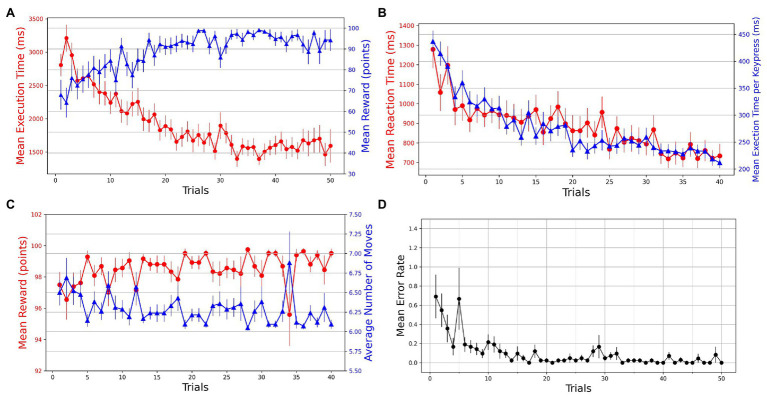
Trial-by-trial course of performance improvement in Mixed-SG condition. The bars on the plot data-points denote standard error in measurement. **(A)** Evolution of learning behavior in the task. Mean execution time and mean reward across trials—averaged over both successful and error trials. **(B)** Mean reaction time and normalized execution time in successful trials. **(C)** Mean reward and average number of moves in successful trials. **(D)** Mean error rates in successful trials.

A within-subjects repeated-measures ANOVA was used to test for the effect of practice (trials) on different behavioral measures. A series of Wilcoxon signed-rank tests was performed on various measures to test for the transfer of learning in Experiment-2. Repeated-measures ANOVA was used to probe any KM-specific or SG-specific effects on the performance. The first 20 successful trials of each SG condition were considered for analysis. Mean execution times and reaction times for a total of 40 successful trials were plotted against the trials. We observe that with practice, the participants become more accurate and efficient in performing GST on the Mixed-SG condition (see Figure 4A). Both the execution time and reaction time, as dependent measures of performance, decrease with practice (see Figure 4B). The learning is evident by the decrease in the mean execution time from 2,854 ms (SD = 971) in the first successful trial to 1,298 ms (SD = 428) in the last successful trial. And the mean reaction time decreased from 1,278 ms (SD = 623) to 733 ms (SD = 403). To evaluate whether the change across the trials is statistically different, we performed a non-parametric Friedman test of differences among repeated measures (within-subjects) for trials 1 through 40. We observed a significant effect of trials on the mean execution time [$\chi^{2}\left(39\right)=423.35,p<0.001$] as well as the mean reaction time [$\chi^{2}\left(39\right)=210.40,p<0.001$]. A Friedman test also indicated a significant effect of trials [$\chi^{2}\left(39\right)=469.06,p<0.001$] on normalized execution time. The mean reward scores improved from 97.50 (SD = 5.325) in the first trial to 99.52 (SD = 1.851) in the last trial. The effect of trials was significant on the mean reward obtained [$\chi^{2}\left(39\right)=61.96,p=0.011$; see Figure 4C]. The average number of moves required to reach the goal position decreased from 6.50 (SD = 1.065) to 6.095 (SD = 0.370) with practice. A Friedman test indicated a significant effect of trials on the average number of moves [$\chi^{2}\left(39\right)=61.96,p=0.011$; see Figure 4C). A Friedman test on mean execution time in optimally successful trials rendered a significant effect [$\chi^{2}\left(39\right)=191.42,p<0.001$] of trials. The mean error rates were computed by averaging the participant error rates while preserving the trial order. A steady decrease in error rates is observed with practice (see Figure 4D).

Additionally, we examined if the performance in the Mixed-SG condition was particular to KM. We performed 2 (KM: 1 and 2) × 40 (Trials: 1–40) mixed repeated-measures analysis of variance (ANOVA) on normalized execution time with KM as a between-subject factor and the trials as a within-subject factor. A Greenhouse-Geisser correction was applied when the ANOVA assumptions were violated. The ANOVA results suggested a significant main effect of trials [$F\left(13.09,523.48\right)=17.326,p<0.001,\eta_{p}^{2}=0.302$], indicating that the normalized execution time varies across the trials. A non-significant main effect of KM [$F\left(1,40\right)=0.782,p=0.382,\eta_{p}^{2}=0.019$] indicated that the normalized execution times are not different for the two KM. Moreover, a non-significant Trial × KM [$F\left(13.09,523.48\right)=1.325,p=0.193,\eta_{p}^{2}=0.032$] interaction suggested that the variation in normalized execution time across the trials is not dependent on KM.

Since the participants used the same KM assignment as that in the Single-SG condition, we anticipate the transfer of learning to occur from the Single-SG condition to the Mixed-SG condition. We analyzed behavioral measures such as error rate, reward, and execution time for the first trial in both conditions to probe for transfer effects. The mean error rate improved from 2.857 (SD = 2.859) in the Single-SG condition to 0.690 (SD = 1.473) in the Mixed-SG condition. A Wilcoxon signed-rank test comparing the error rates during the first trials of both conditions reported significant differences (df=41,Z=508.00,p<0.001). The mean reward score improved from 20.952 (SD = 40.714) in the Single-SG condition to 67.976 (SD = 46.224) in the Mixed-SG condition. A Wilcoxon signed-rank test revealed that the mean reward obtained is significantly higher (df=41,Z=36.00,p<0.001) for the initial trial in the Mixed-SG condition as compared to the first trial in the Single-SG condition. Similarly, the mean execution time improved from 3,743 (SD = 1,380) ms to 2,807 (SD = 1,083) ms due to the transfer effects. A Wilcoxon signed-rank test suggested that the mean execution time for the first trial in the Single-SG condition is significantly different (df=41,Z=743.00,p<0.001) as compared to the first trial of the Mixed-SG condition.

The Single-SG condition does not require participants to employ all three keys to reach the goal position. In both the KM groups, the participants only needed keys 4 and 5 to build the trajectory from the start to the goal position. Therefore, in both the KM groups, at the end of the Single-SG condition, the participants are highly trained with the response effects for two (keys 4 and 5) of the three keys. In the Mixed-SG condition, condition SG1 requires using keys 5 and 6 to build an optimal trajectory, whereas condition SG2 requires keys 4 and 5 to navigate to the goal position. Since the participants are highly trained on response-effect contingencies for keys 4 and 5 but not for key 6, the differential amount of practice may benefit performance on condition SG2 (employing keys 4 and 5) but not condition SG1 (employing keys 5 and 6). Thus, one can argue that performance will be influenced by a differential amount of practice based on SG conditions in the Mixed-SG condition. To probe this, we analyzed the effect of SG on execution time in the Mixed-SG condition. We performed 2 (SG: 1 and 2) × 20 (successful trials: 1–20) repeated-measures ANOVA on the mean execution time for each KM. A Greenhouse-Geisser correction was applied when the ANOVA assumptions were violated. For KM1, the ANOVA results reported a main effect of trials [$F\left(4.875,112.123\right)=27.583,p<0.001,\eta^{2}=0.402$], suggesting practice-driven learning. The test reported a non-significant main effect of SG [$F\left(1,23\right)=3.915,p=0.060,\eta^{2}=0.006$] and Trial × SG interaction [$F\left(6.983,160.601\right)=1.081,p=0.378,\eta^{2}=0.010$]. Similarly, for KM2, the ANOVA results reported the main effect of trials [$F\left(19,323\right)=13.192,p<0.001,\eta^{2}=0.263$], suggesting practice-driven performance improvements. It suggested a non-significant main effect of SG [$F\left(1,17\right)=0.035,p=0.854,\eta^{2}=0.0001$] and Trial × SG interaction [$F\left(19,323\right)=1.204,p=0.252,\eta^{2}=0.023$]. The results suggest that the execution time is not different for the two SGs in the Mixed-SG condition in both the KM groups. Therefore, the performance is not influenced by the differential amount of practice based on SG conditions in the Mixed-SG condition.

### Discussion

The randomized and mixed order of SG conditions in Experiment-2 minimized the trajectory-specific performance improvements that occur due to the repeated execution of the keypress sequences. Significant performance improvements were observed for normalized execution time in successful trials and execution time in optimally-successful trials. Other performance measures such as reward and reaction time also improved with practice. This efficient performance of GST on new and randomly-ordered SG conditions can be attributed to the ability to use a previously learned KM-specific internal model for planning navigation strategies. As the learned KM relations can be successfully applied to new SG conditions, the participants could generalize the learning from the Single-SG condition to the Mixed-SG condition. The positive transfer effects support the idea of cognitive learning because the participants cannot simply transfer a learned motor sequence in the Mixed-SG condition.

In both the KM groups, the Single-SG condition employed only two (keys 4 and 5) of the three possible cursor movements to construct an optimal trajectory. One can raise the question that the transfer of learning would be better on the novel SG condition that employed the same practiced keys (namely, condition SG2) compared to the other novel SG condition (namely, condition SG1) in Experiment-2. However, the analysis revealed no difference in performance in execution time in both the SG conditions. This suggests that the transfer of the internal model related to the KM is not contingent on the specific keys that are practiced in various SG conditions.

## General Discussion

We investigated the nature of learning in internally-guided sequencing. We argued that GST-like grid-navigation tasks are exemplars of such a paradigm and hypothesized the role of motor and cognitive learning processes in learning in GST. We proposed a novel use of GST in two behavioral experiments to this end. In Experiment-1 (Single-SG condition), we investigated the progressive nature of learning, as evidenced by improvements in various behavioral measures. We provide evidence for the role of trajectory-specific motor learning in GST by showing the effect of trials on execution time in the Single-SG condition. The performance degradation on the introduction of a visuomotor rotation trial suggests that the learning in GST involves the acquisition of a motor program and therefore, it cannot be solely attributed to the general motor improvements. In Experiment-2 (Mixed-SG condition), we provide evidence for the role of KM-specific, trajectory-independent learning in GST. The transfer-related performance improvements in the Mixed-SG condition provide evidence for the acquisition of a KM-specific internal model that translates as cognitive learning in GST.

### General Stages of Learning in GST

Improvements in various behavioral measurements such as execution time, reaction time, and reward score indicate learning in GST (see Figure 2). In the Single-SG condition, the participants initially tried to learn the possible movement directions and the corresponding key-map (KM) by trial and error. As the participants became familiar with the association between keypresses and corresponding cursor movements, they learn the effects of their responses. In further attempts, using the learned KM, the participants execute the keypresses to move the cursor in the direction of the target. Further practice enables them to plan simple and optimal navigation strategies to reach the goal. In the late phase, the repeated execution of the optimal trajectory drives performance improvements due to motor learning. We anticipate the role of motor chunking, due to which the planned trajectory to the goal position is segmented into sub-sequences of individual motor actions (keypresses). The late stage of practice would be characterized by an “automatic” mode of execution with reduced cognitive and attentional demands. Motor chunking would enable the participants to perform the sequence as a whole without relying on individual response-effect contingencies. The practice-driven performance improvements in GST due to chunking are investigated in another study (Bera et al., 2021).

The trajectory planning was guided by feedback from the reward score and the number of moves (see Figure 2A). The reward feedback gives a measure of the optimality of the trajectory followed. A steep decrease in the number of error trials (error rates) is observed after the first successful trial (see Figure 2D). Further practice enabled planning of optimal trajectories, as evident from the increase in mean reward. The participants quickly hit the reward ceiling (reward = 100) within 10–15 trials, implying that they have learned to navigate optimally. After a substantial amount of practice, as the KM model and SG trajectories are thoroughly learned, the (reward) feedback became less consequential for task accuracy (see Figure 2C). Nevertheless, we saw a further performance improvement in normalized execution time (task speed) of successful trials (see Figure 2B). To control the number of moves over which the execution time is computed in successful trials, we performed the normalized execution time analysis. A statistically significant improvement in normalized execution time shows growing expertise in performing the sequence. While multiple optimal trajectories are possible for a given SG position, the performance improvements due to repeated execution of the same trajectory can be attributed to motor learning. Therefore, we also performed the execution time analysis with a control on the number of moves and the sequential keypresses of the trajectory traversed. A statistically significant improvement in execution time confirms the role of motor learning due to repeated execution of the same trajectories.

### Cognitive Aspects of Internally-Guided Sequencing

In addition to motor learning, the performance in internally-guided paradigms is also contingent on the ability to plan the sequence of actions efficiently. In GST, determining the sequence of keypress execution corresponds to planning a trajectory from the start to the goal position. Such planning and trajectory-generation are analogous to the goal-directed behavior in the knight’s tour on a chessboard. To reach a given goal position on the chessboard, goal-directed planning is employed to generate an optimal sequence of moves (analogous to a trajectory in GST) using an internal map based on possible movement directions of a knight (similar to a KM in GST). In both cases, the conceived reach pattern used for planning trajectories is KM-specific. The acquisition of such a KM-specific internal model helps in planning trajectories and amounts to cognitive learning in GST.

Therefore, we hypothesized that cognitive learning processes contribute to the learning in GST. The role of cognitive learning could be confirmed if the participants can generalize the learning from a learned SG condition to other novel SG conditions. To test this, we performed Experiment-2, where we asked the participants to perform GST on randomized and mixed order of novel SG positions using the learned KM from Experiment-1. The transfer-related performance improvements in various behavioral measures confirm the role of cognitive learning. Since the participants cannot readily utilize the previously learned motor sequences on novel SG conditions, the transfer-related performance gains occur due to a trajectory-independent learning component. The two experiments are not independent because the same participants employed the same KM. Therefore, the improvements suggest that the KM-specific internal model is acquired and transferred from the Single-SG condition to facilitate efficient trajectory planning in the Mixed-SG condition. The acquisition of an internal model involves learning the KM relations between the possible cursor movements and the keypress buttons. This internal model is employed while planning the trajectories to generate a new sequence of keypresses that can be executed to solve a novel SG condition. Therefore, the cognitive component in GST is a form of the trajectory-independent learning process and it involves the acquisition of a KM-specific internal model.

**Figure 5:**
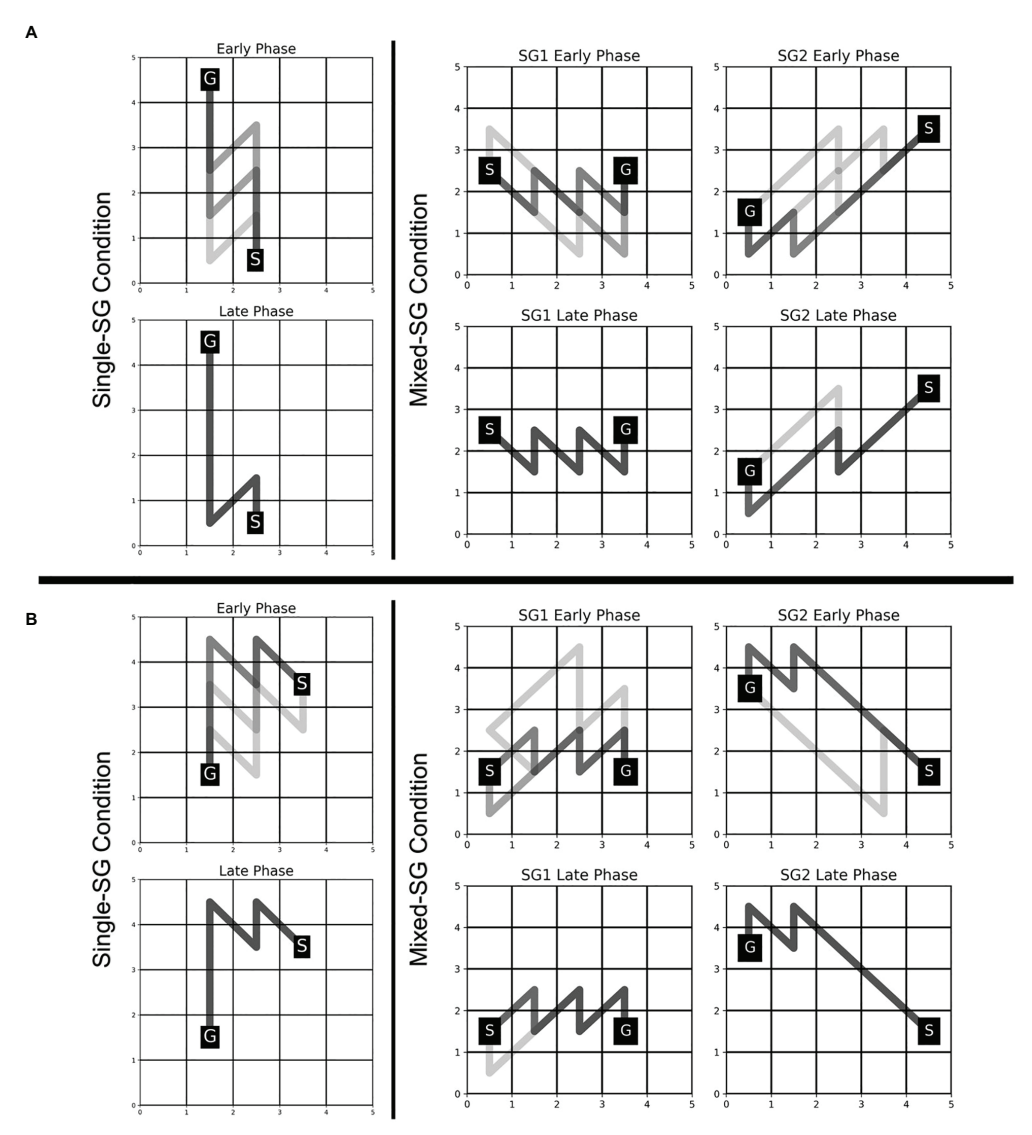
Evolution of trajectories in Single-SG and Mixed-SG condition in two representative participants—MD **(A)** and AS **(B)**. Participants MD and AS are assigned key-maps KM1 and KM2, respectively. The comparison of trajectories in early vs. late phase is shown. The early and late phase correspond to the first and last five successful trials, respectively, in each condition. A darker trajectory shade denotes more frequented trajectory.

The participants were able to employ the learned KM (from the Single-SG condition) to plan trajectories to the goal position with minimal failed attempts, as evident from a significant decrease in the error rate from 2.857 in the Single-SG condition to 0.690 in the Mixed-SG condition. The error rates denote the average number of error trials attempted to complete each successful trial. The fraction of participants who performed the first trial without any errors increased from 21% in the Single-SG condition to 69% in the Mixed-SG condition. Moreover, a qualitative examination of the evolution of trajectories in the early and late phases of the Mixed-SG condition suggests the role of a KM-specific learning component in GST. It is apparent from Figure 5 that the participants employed the learned KM-specific internal model to improvise on non-optimal trajectories in the early phase. Thus, the late phase is characterized by optimal trajectory planning and increased trajectory density.

This account of transfer of learning is also corroborated by improvements in other behavioral measures such as the mean execution time and average reward score. The mean execution time decreased from 3,743 to 2,807 ms as the participants were able to quickly plan trajectories using the acquired KM on novel SG conditions. The mean reward score also improved from 20.952 in the Single-SG condition to 67.976 in the Mixed-SG condition. In summary, these results suggest that the participants are faster, more accurate, and quickly discover the optimal trajectory in the Mixed-SG condition as compared to the Single-SG condition. It suggests a key contribution of transfer of the acquired key-map from the Single-SG condition to the Mixed-SG condition.

In addition, we observed a significant effect of practice on the reaction time in Single-SG and Mixed-SG conditions (see Figures 2B, 4B). This result is rather intriguing because no improvements in reaction time were expected in line with the previous findings (Fermin et al., 2010). The reaction time denotes the latency of the first keypress, reflecting the time cost of pre-planning the whole trajectory from the start to the goal position. A steady decrease in reaction time implies that the participants become more adept at using the previously acquired KM-specific internal model to plan trajectories with practice. The reaction time trend provides additional evidence for the involvement of cognitive learning in GST.

**Figure 6:**
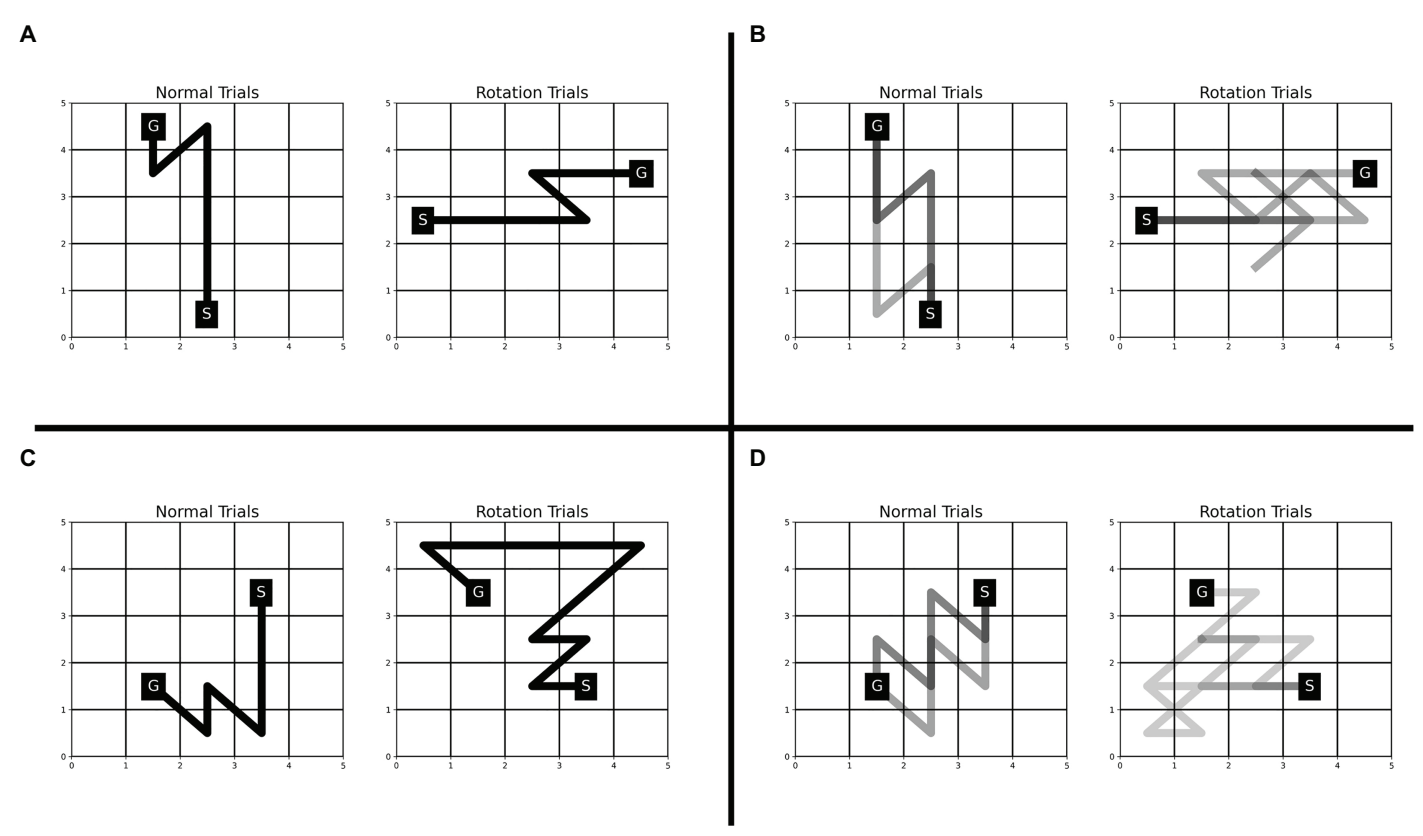
Comparison of trajectories traversed during normal and rotation trials in Experiment-1 for four representative participants—JK **(A)**, LM **(B)**, RS **(C)**, and CP **(D)**. The trajectories for all the rotation trials are plotted for each participant. The number of trajectories plotted for the normal condition is matched with that from the rotation condition. The number of trajectories (or trials) plotted for participants JK, LM, RS, and CP is 1, 3, 1, and 5, respectively. A darker trajectory shade denotes more frequented trajectory.

The Single-SG condition involved a rotation trial. One possible way to complete the rotation trial efficiently, would be to execute the learned sequence of keypresses after performing a mental rotation of the KM and SG positions to “undo” the rotation. If participants employed this strategy, we would anticipate that the reaction times increase but not the execution times. However, we observed an increase in execution time and reaction time even on multiple attempts on the rotation trial (see Figure 3). This indicates that the participants may have attempted the rotation trial as a novel KM-SG condition. Consequently, the execution time increased due to the additional time cost of planning trajectories using a novel KM. The performance degradation is also evident from other behavioral measures such as reward score and error rates. We further examined the differences in the trajectories traversed in the normal and rotation condition (see Figure 6). We observed many qualitative differences between trajectories traversed in normal and rotation conditions, irrespective of the number of attempts on the rotation trial. Overall, the results in the rotation trials suggest that the trajectory-specific motor program learned in the normal condition could not be transferred to the rotation condition successfully.

### Theoretical Perspectives on Internally-Guided Sequencing

Fermin et al. (2010) provide evidence for model-based action planning in GST by demonstrating that the participants benefit from previously learned state transition models (or KM) if an additional delay is given before the start of the movements. For learned KM, such a delay would favor model-based planning using internal simulations of sequential action selection. In our case, such acquisition of the internal model is evidenced by the ability to efficiently navigate in a randomly-ordered mixed-SG condition where trajectory/sequence-specific learning due to visuomotor associations is minimized. While Fermin and colleagues established the progressive nature of learning in different stages based on model-based and model-free action selection strategies, we examined the behavior in GST in terms of cognitive-motor dichotomy in internally-guided sequencing. We provide evidence to establish the role of cognitive as well as motor learning in GST. Numerous studies have tried to reconcile the computational (model-based vs. model-free) and behavioral (cognitive vs. motor) perspectives in understanding the distinct, parallel processes involved in motor learning (Keele et al., 2003; Wolpert et al., 2011; Dezfouli and Balleine, 2012; Wolpert and Landy, 2012; Savalia et al., 2016; McDougle and Taylor, 2019). In line with Fermin et al. (2010), we show evidence for the role of the cognitive learning process as part of goal-directed, model-based action planning in GST. The implications of our findings are two-fold—while establishing the role of cognitive and motor processes in the non-trivial planning and sequence execution in GST, we call for a renewed interest in understanding a class of practical, internally-guided motor sequence learning tasks. In sum, sequencing behavior in GST involves both general motor learning and acquisition of an internal model. Learning the association between the movements and the corresponding keypresses allows for the acquisition of sequence as participants learn to “react” appropriately and efficiently to the motor intention or plan as circumscribed by the KM-specific internal model. General motor learning results in quick and efficient performance with repeated execution of finger movements in response to visual cues. The performance improvements due to motor learning may be driven by motor chunking. In GST-like internally-guided tasks, practice-driven motor learning is constrained by a goal-directed internal plan of sequential actions. The internal model in GST involves the acquisition of a general structure or organization which guides the sequential order of keypress execution. A salient feature of such paradigms is that motor learning is influenced by the structure and organization of practiced sequences. It is guided internally and not externally imposed. Consequently, internally-guided paradigms involve developing internal representations for both the response-effect mappings for KM and the sequence of keypresses (trajectory) to reach the goal. These internal representations are subject-specific even when the participants were using the same KM on a similar SG condition. Our account is again in line with the previous studies on the ideomotor framework of voluntary action control. The action-effect (or R-E) bindings emerge during action planning, integrating components of the forward and inverse models of motor control (Ziessler et al., 2004; Nattkemper et al., 2010).

GST involves the role of interleaved cognitive and motor learning components. This parallel trajectory planning and motor learning induce a natural duality in the task. We speculate the role of working memory and visuospatial attention in GST. The task involves divided attention where information such as KM and the current trajectory is actively maintained online in the working memory. In contrast, SG information in the visual buffer helps in directing the cursor towards the goal position. The executive control inhibits the natural tendency of executing a response to generate the appropriate sequence of keypresses, given the constraints of the KM-specific internal plan. The early practice phase would be characterized by high attentional and cognitive demands as the participants learn response-effect mapping for the KM. With trial and error, as they learn to move the cursor towards the goal, the visuospatial attention and working memory are actively engaged to strategize navigation to the goal position. Further practice allows for optimizing the trajectories to reach the goal position in an optimal number of moves. Once an optimal path is discovered in the late practice phase, the performance improvements occur predominantly due to motor learning. We speculate that motor chunking characterizes automatic and habitual control with reduced attention.

Grid-sailing task is a simple canonical paradigm that does not require any complicated experimental setting, yet it offers rich insights into the planning and sequencing behavior. Unlike other discrete sequencing tasks such as SRT, DSP, or *m* × *n* task, GST involves learning self-generated motor sequences. In GST, the sequential keypresses are not guided by an external series of stimuli but are instead self-initiated by a KM-specific internal model. The behavior in GST can be organized into the “planning” and “executing” phases. These distinct phases enable natural dissociation of cognitive and motor strategies involved in internally-guided sequence learning. This is a unique and helpful characteristic of GST that can be leveraged to investigate the role of different learning processes involved in internally-guided sequencing. The cognitive phase in GST can be distinctly associated with acquisition of the trajectory-independent and KM-specific internal model, which is employed while navigating the grid. The learned KM could also enable a selective transfer of the learned model to other tasks where the KM is compatible. Therefore, GST can also be used to study skill transfer and related behavioral phenomena. Moreover, the GST task paradigm affords variations in different aspects. The GST instances can differ in various factors such as grid-size, start-goal (SG) positions used, KMs associated with the task, and the number of cursor movement directions. Owing to many possible variations in the GST paradigm, the instances cover a broad spectrum of grid-navigation tasks that vary across aspects such as the difficulty of solving, execution time required, and cognitive effort demanded—providing reasonable experimental control that is necessary to study different factors involved in sequence learning tasks.

## Conclusion

Using GST as an exemplar paradigm of the grid-navigation task, we provide evidence for cognitive and motor learning in internally-guided sequencing. In the Single-SG condition, we show that the overall learning in GST is evident from the performance improvements in various behavioral measures. We further show increasing dexterity on repeated execution of the same trajectories as evidence for motor learning. A rotation was introduced in the Single-SG condition to probe if the performance improvements can be solely attributed to general motor learning. The performance degradation on the rotation trial suggests that the learning is not occurring only due to general motor learning. We further hypothesized that cognitive learning contributes to the learning in GST. The role of such a cognitive learning process is confirmed by showing transfer-related performance improvements on the randomized and mixed order of previously unseen SG conditions in the Mixed-SG condition. We show that the participants generalize and transfer a KM-specific internal model from the Single-SG condition to the Mixed-SG condition. We further advance the use of grid-navigations tasks in investigating internally-guided sequencing.

## Limitations and Future Directions

The experimental paradigm can be modified to incorporate a within-subject control over the executed trajectories to probe motor chunking. Future work can also attempt to examine the differences in trajectory traversals in different conditions statistically. For example, a hypothetical measure of trajectory density can be used to understand the evolution of trajectories in Single-SG conditions. A categorical comparison of the trajectory features in Single-SG and Mixed-SG conditions can reveal further evidence for the “transfer” of KM-specific learning. The role of KM-specific learning can be further validated by introducing a new KM on the same SG conditions. Future studies can also employ a retention task by extending the experimental task over a period of days to dissociate the cognitive and motor learning in GST. We anticipate that the KM-specific internal model will be retained longer than the fine-tuned motor movements specific to the trajectory. Consequently, we can expect to see that the participants quickly recall the learned KM and perform better than they had initially performed at the beginning of the task.

## Data Availability Statement

The raw data supporting the conclusions of this article will be made available by the corresponding author on reasonable request.

## Ethics Statement

The studies involving human participants were reviewed and approved by Institute Review Board (IRB), IIIT-Hyderabad, India. Written informed consent to participate in this study was provided by the participants, and where necessary, the participants’ legal guardian/next of kin.

## Author Contributions

KB and RB conceptualized the study. KB and AS designed the experiment. KB collected the data. KB, AS, and RB contributed to data analysis, interpretation, and manuscript writing. All authors contributed to the article and approved the submitted version.

### Conflict of Interest

The authors declare that the research was conducted in the absence of any commercial or financial relationships that could be construed as a potential conflict of interest.

## Footnotes

^1^Retrieved from https://www.pygame.org

## References

[ref1] Abrahamse E. L. Ruitenberg M. F. L. de Kleine E. Verwey W. B. (2013). Control of automated behavior: insights from the discrete sequence production task. Front. Hum. Neurosci. 7:82. 10.3389/fnhum.2013.00082, PMID: 23515430 PMC3601300

[ref2] Bapi R. S. Doya K. Harner A. M. (2000). Evidence for effector independent and dependent representations and their differential time course of acquisition during motor sequence learning. Exp. Brain Res. 132, 149–162. 10.1007/s002219900332, PMID: 10853941

[ref3] Bapi R. S. Miyapuram K. P. Graydon F. X. Doya K. (2006). fMRI investigation of cortical and subcortical networks in the learning of abstract and effector-specific representations of motor sequences. NeuroImage 32, 714–727. 10.1016/j.neuroimage.2006.04.205, PMID: 16798015

[ref4] Bera K. Shukla A. Bapi R. S. (2021). Motor chunking in internally guided sequencing. Brain Sci. 11:292. 10.3390/brainsci11030292, PMID: 33652707 PMC7996945

[ref5] Clegg B. A. DiGirolamo G. J. Keele S. W. (1998). Sequence learning. Trends Cogn. Sci. 2, 275–281. 10.1016/S1364-6613(98)01202-9 21227209

[ref6] Dezfouli A. Balleine B. W. (2012). Habits, action sequences and reinforcement learning: habits and action sequences. Eur. J. Neurosci. 35, 1036–1051. 10.1111/j.1460-9568.2012.08050.x, PMID: 22487034 PMC3325518

[ref7] Doya K. (2000). Complementary roles of basal ganglia and cerebellum in learning and motor control. Curr. Opin. Neurobiol. 10, 732–739. 10.1016/S0959-4388(00)00153-7, PMID: 11240282

[ref8] Drucker J. H. Sathian K. Crosson B. Krishnamurthy V. McGregor K. M. Bozzorg A.. (2019). Internally guided lower limb movement recruits compensatory cerebellar activity in people with Parkinson’s disease. Front. Neurol. 10:537. 10.3389/fneur.2019.00537, PMID: 31231297 PMC6566131

[ref9] Fermin A. Yoshida T. Ito M. Yoshimoto J. Doya K. (2010). Evidence for model-based action planning in a sequential finger movement task. J. Mot. Behav. 42, 371–379. 10.1080/00222895.2010.526467, PMID: 21184355

[ref10] Fermin A. S. R. Yoshida T. Yoshimoto J. Ito M. Tanaka S. C. Doya K. (2016). Model-based action planning involves cortico-cerebellar and basal ganglia networks. Sci. Rep. 6:31378. 10.1038/srep31378, PMID: 27539554 PMC4990901

[ref11] Fitts P. M. Posner M. I. (1967). Human performance. Belmont, Calif.: Brooks/Cole Pub. Co.

[ref12] Ghilardi M. F. Moisello C. Silvestri G. Ghez C. Krakauer J. W. (2009). Learning of a sequential motor skill comprises explicit and implicit components that consolidate differently. J. Neurophysiol. 101, 2218–2229. 10.1152/jn.01138.2007, PMID: 19073794 PMC2681421

[ref13] Gowen E. Miall R. C. (2007). Differentiation between external and internal cuing: an fMRI study comparing tracing with drawing. NeuroImage 36, 396–410. 10.1016/j.neuroimage.2007.03.005, PMID: 17448689 PMC2570483

[ref14] Greenwald A. G. (1970). Sensory feedback mechanisms in performance control: with special reference to the ideo-motor mechanism. Psychol. Rev. 77, 73–99. 10.1037/h0028689, PMID: 5454129

[ref15] Haibach P. Reid G. Douglas C. (2017). Motor learning and development. 2nd Edn. Champaign, IL, US: Human Kinetics.

[ref16] Hebb D. O. (1961). “Distinctive features of learning in the higher animal” in Brain mechanisms and learning. ed. Delafresnaye J. F. (Oxford: Blackwell), 37–46.

[ref17] Herwig A. Waszak F. (2009). Intention and attention in ideomotor learning. Q. J. Exp. Psychol. 62, 219–227. 10.1080/17470210802373290, PMID: 18932052

[ref18] Hikosaka O. Rand M. K. Miyachi S. Miyashita K. (1995). Learning of sequential movements in the monkey: process of learning and retention of memory. J. Neurophysiol. 74, 1652–1661. 10.1152/jn.1995.74.4.1652, PMID: 8989401

[ref19] Hommel B. (2003). “Acquisition and control of voluntary action” in Voluntary action: Brains, minds, and sociality. eds. Maasen S. Prinz W. Roth G. (Oxford: Oxford University Press), 34–48.

[ref20] Hommel B. Müsseler J. Aschersleben G. Prinz W. (2001). The theory of event coding (TEC): a framework for perception and action planning. Behav. Brain Sci. 24, 849–878. 10.1017/S0140525X01000103, PMID: 12239891

[ref21] JASP Team (2020). *JASP (Version 0.14.1) [Computer software]*. Available at: https://jasp-stats.org/ (Accessed February 28, 2021).

[ref22] Jueptner M. (1998). A review of differences between basal ganglia and cerebellar control of movements as revealed by functional imaging studies. Brain 121, 1437–1449. 10.1093/brain/121.8.1437, PMID: 9712006

[ref23] Jueptner J. Jueptner M. Jenkins I. H. Brooks D. J. Frackowiak R. S. J. Passingham R. E. (1996). The sensory guidance of movement: a comparison of the cerebellum and basal ganglia. Exp. Brain Res. 112, 462–474. 10.1007/BF00227952, PMID: 9007548

[ref24] Keele S. W. Ivry R. Mayr U. Hazeltine E. Heuer H. (2003). The cognitive and neural architecture of sequence representation. Psychol. Rev. 110, 316–339. 10.1037/0033-295X.110.2.316, PMID: 12747526

[ref25] Lashley K. S. (1951). “The problem of serial order in behavior” in Cerebral mechanisms in behavior. ed. Jeffress L. A. (New York: Wiley), 112–131.

[ref26] Logan G. D. (1988). Toward an instance theory of automatization. Psychol. Rev. 95, 492–527. 10.1037/0033-295X.95.4.492

[ref27] McDougle S. D. Taylor J. A. (2019). Dissociable cognitive strategies for sensorimotor learning. Nat. Commun. 10, 40. 10.1038/s41467-018-07941-0, PMID: 30604759 PMC6318272

[ref28] Nattkemper D. Ziessler M. Frensch P. A. (2010). Binding in voluntary action control. Neurosci. Biobehav. Rev. 34, 1092–1101. 10.1016/j.neubiorev.2009.12.013, PMID: 20036685

[ref29] Newell K. M. (1991). Motor skill acquisition. Annu. Rev. Psychol. 42, 213–237. 10.1146/annurev.ps.42.020191.001241, PMID: 2018394

[ref30] Nissen M. J. Bullemer P. (1987). Attentional requirements of learning: evidence from performance measures. Cogn. Psychol. 19, 1–32. 10.1016/0010-0285(87)90002-8

[ref31] Penhune V. B. Steele C. J. (2012). Parallel contributions of cerebellar, striatal and M1 mechanisms to motor sequence learning. Behav. Brain Res. 226, 579–591. 10.1016/j.bbr.2011.09.044, PMID: 22004979

[ref32] Prinz W. (1997). Perception and action planning. Eur. J. Cogn. Psychol. 9, 129–154. 10.1080/713752551

[ref33] Robertson E. M. (2007). The serial reaction time task: implicit motor skill learning? J. Neurosci. 27, 10073–10075. 10.1523/JNEUROSCI.2747-07.2007, PMID: 17881512 PMC6672677

[ref34] Sakai K. Kitaguchi K. Hikosaka O. (2003). Chunking during human visuomotor sequence learning. Exp. Brain Res. 152, 229–242. 10.1007/s00221-003-1548-8, PMID: 12879170

[ref35] Savalia T. Shukla A. Bapi R. S. (2016). A unified theoretical framework for cognitive sequencing. Front. Psychol. 7:1821. 10.3389/fpsyg.2016.01821, PMID: 27917146 PMC5114455

[ref36] Schmidt R. A. Lee T. D. Winstein C. J. Wulf G. Zelaznik H. N. (2019). Motor control and learning: A behavioral emphasis. Champaign, IL: Human Kinetics.

[ref37] van Donkelaar P. Stein J. F. Passingham R. E. Miall R. C. (1999). Neuronal activity in the primate motor thalamus during visually triggered and internally generated limb movements. J. Neurophysiol. 82, 934–945. 10.1152/jn.1999.82.2.934, PMID: 10444688

[ref38] Verwey W. B. (2001). Concatenating familiar movement sequences: the versatile cognitive processor. Acta Psychol. 106, 69–95. 10.1016/S0001-6918(00)00027-5 11256340

[ref39] Verwey W. B. Shea C. H. Wright D. L. (2015). A cognitive framework for explaining serial processing and sequence execution strategies. Psychon. Bull. Rev. 22, 54–77. 10.3758/s13423-014-0773-4, PMID: 25421407

[ref40] Willingham D. B. (1999). Implicit motor sequence learning is not purely perceptual. Mem. Cogn. 27, 561–572. 10.3758/BF03211549, PMID: 10355244

[ref41] Wolpert D. M. Diedrichsen J. Flanagan J. R. (2011). Principles of sensorimotor learning. Nat. Rev. Neurosci. 12, 739–751. 10.1038/nrn3112, PMID: 22033537

[ref42] Wolpert D. M. Landy M. S. (2012). Motor control is decision-making. Curr. Opin. Neurobiol. 22, 996–1003. 10.1016/j.conb.2012.05.003, PMID: 22647641 PMC3434279

[ref43] Ziessler M. Nattkemper D. Frensch P. A. (2004). The role of anticipation and intention in the learning of effects of self-performed actions. Psychol. Res. 68, 163–175. 10.1007/s00426-003-0153-6 14634810

